# Radical resection and sentinel lymph node evaluation of mammary-like adenocarcinoma of the vulva (MLAV) with somatic BRCA1 mutation

**DOI:** 10.1016/j.gore.2025.101957

**Published:** 2025-09-19

**Authors:** Lilla Markel, Lidys Rivera, A. Ordobazari, Ardeshir Hakam, Wilfredo Lorenzo, Mitchel S. Hoffman, Robert M. Wenham, Monica Avila

**Affiliations:** a USF Department of Obstetrics and Gynecology, Tampa General Hospital, Tampa, USA; b Department of Pathology, Moffitt Cancer Center, Tampa, FL, USA; c Florida Woman Care, St. Petersburg, Florida, USA; d Division of Gynecologic Oncology, Department of Surgery, Moffitt Cancer Center, Tampa, USA

**Keywords:** Mammary-like gland adenocarcinoma of the vulva, BRCA1, Sentinel node biopsy

## Abstract

**Background:**

Mammary-like gland adenocarcinoma of the vulva is a rare and aggressive form of vulvar cancer with histopathologic resemblance to breast cancer. This rare type of vulvar cancer develops from primary metaplasia of mammary-like anogenital glands, more recently discovered anatomic components of the anogenital region.

**Case presentation:**

We present a 77-year-old African-American female with a six-month history of painless, left lower vulvar lesion referred to our cancer center following confirmatory biopsy of invasive mammary type adenocarcinoma. Preoperative PET CT demonstrating focal avidity (SUV 7.8) in the vulvar region with no evidence of metastatic disease. She underwent exam under anesthesia, left radical vulvar excision with primary closure, left inguinal sentinel lymph node mapping and biopsy for a TNM stage of T2N0M0 and vulvar FIGO stage IB grade 2. Somatic testing was significant for a BRCA1 mutation (variant p.S770, c.2309C>G).

**Conclusion:**

Mammary-like gland adenocarcinoma of the vulva is a rare but aggressive neoplasm that presents diagnostic challenges due to its resemblance to breast carcinoma. Radical resection and sentinel lymph node assessment is feasible. Melanocytic skin types pose a unique set of differences regarding detection, diagnosis and potential genomic variations such as BRCA1 mutation in our patient. Genetic and genomic assessments should be considered to further guide optimal therapeutic options.

## Highlights

• MLAV arises in the vulva from mammary-like anogenital glands histologically similar to breast cancer.
• Sentinel lymph node mapping and biopsies with indocyanine green (ICG) dye is feasible for staging MLAV.
• Somatic and germline testing should be considered as part of the work up for MLAV.
• Pathogenic somatic variants like BRCA1 can potentially direct targeted therapies.

## 1 Introduction

Mammary-like gland adenocarcinoma of the vulva is a rare and aggressive form of vulvar cancer with histopathologic resemblance to breast cancer. It was originally theorized that these cancers originate from ectopic breast tissue, located anywhere along the embryonic milk line, which extends from the axilla to the groin and includes the vulva (Velanovich, 1995, Mansour et al., 2023). The incidence of ectopic mammary gland tissue is low, about 1–6 % in the general population, with the vulva being one of the most infrequent sites (Velanovich, 1995, Mansour et al., 2023, Lopes et al., 2018). The origin of a vulvar malignancy arising from ectopic breast tissue was founded on morphological similarities, immunohistochemical (IHC) profiles, and comparable aggressive behavior that are now understood to be intrinsic to the anogenital area (Kazakov et al., 2011). It is now thought this rare type of vulvar cancer develops from metaplasia of the mammary-like anogenital glands, which are more recently discovered anatomic components of the anogenital region (Abbott and Ahmed, 2006, van der Putte, 1994, Si et al., 2024, Niakan et al., 2021, Morais et al., 2022). These glands feature epithelial structures with either eccrine or apocrine differentiation and have the potential to develop both benign and malignant lesions (Kazakov et al., 2011). The glands serve similar physiologic function to breast tissue and respond to hormonal stimuli (Morais et al., 2022). IHC analyses have revealed triple positive expression of the estrogen, progesterone and HER2 receptor supporting a mammary-like gland origin of the tumor (Hu and Tiesinga, 2024). ERBB2, PIK3CA, and TP53 have been genes previously associated in this histotype, although mutation type varies (Hu and Tiesinga, 2024, Tessier-Cloutier et al., 2017).

Given the rarity of mammary-like gland adenocarcinoma of the vulva, current knowledge is limited to case reports and there is no established standard of care or targeted therapeutic approach. We present the case of a 77-year-old African-American female diagnosed with mammary-gland like adenocarcinoma of the vulva with micropapillary features.

## 2 Case presentation

**Fig. 1:**
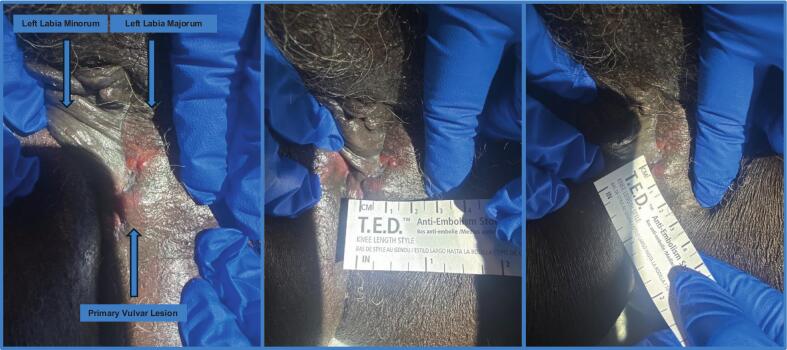
A. Primary Vulvar Lesion: raised, fluctuant and erythematous with superior vesicular extension (left labia minorum is medialized) B. Primary Vulvar Lesion: left to right measurement in cm C. Primary Vulvar Lesion: superior to inferior measurement in cm.

**Fig. 2:**
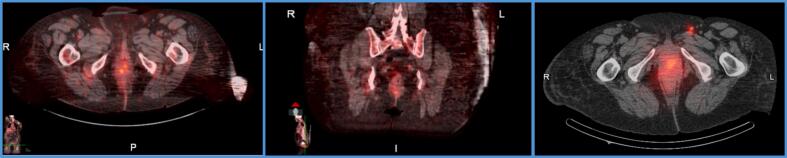
A. Preoperative PET CT: Axial Fused CT Image noting avidity (SUV 7.8) at level of the vulva B. Preoperative PET CT: Coronal Fused CT Image noting avidity at level of the vulva C. Preoperative Spectral PET CT: Axial Fused CT Image following Technetium 99 injection of primary vulvar lesion denoting left inguinal sentinel lymph node.

The patient is a 77-year-old African-American female with a six-month history of painless, left lower vulvar lesion referred to our cancer center following confirmatory biopsy of invasive mammary type adenocarcinoma by her gynecologist. Medical history was significant for multiple myeloma and chronic kidney disease stage 4. Surgical history includes cesarean section, hysterectomy with unilateral oophorectomy, and bilateral knee replacement. No significant family history was reported. Initial physical exam at our institution revealed a 2 × 1 cm left lower, non-ulcerated, fluctuant vulvar lesion (Fig. 1). The biopsy was reported positive for ER, EMA, Bcl-2, CK7, GATA3, while negative for SOX10, Melan-A. HER2 was weakly positive with a score of 1+. She had a preoperative PET CT demonstrating focal avidity (SUV 7.8) in the vulvar region with no evidence of metastatic disease (Fig. 2). Preoperative mammogram was BI-RADS 2.

**Fig. 3:**
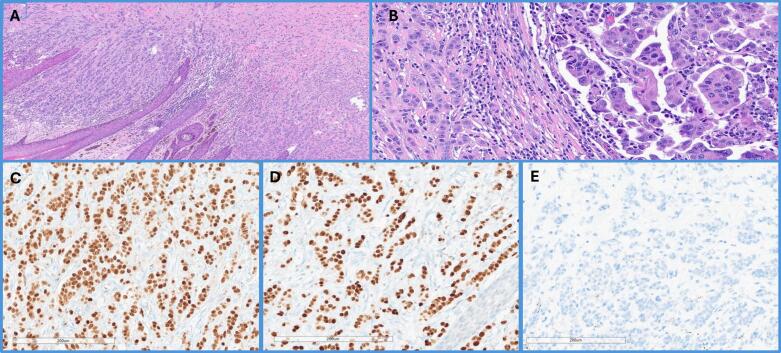
Adenocarcinoma of mammary gland type. A) 4X. H&E. Partial vulvectomy, demonstrating unremarkable squamous epithelium, while tumor cells show an infiltrative growth in the dermis; B) 10X. H&E Adenocarcinoma with multiple foci exhibiting micropapillary features (right), and glandular architecture or in small cords (left). C-E) Immunohistochemistry profile with staining demonstrating positivity for ER (C), and GATA-3 (D), and negativity for mammaglobin (E).

On day of surgery, she underwent spectral PET CT for inguinal sentinel lymph node mapping with Technetium 99 injection at site of primary vulvar lesion (Fig. 2). She underwent exam under anesthesia, left radical vulvar excision with primary closure, left inguinal sentinel lymph node mapping and biopsy. Sentinel lymph node mapping was performed with combination Technetium 99 with gamma detector and Indocyanine green (ICG) dye with immunofluorescence imaging intra-operatively. The vulvar specimen demonstrated atypical epithelial cells with prominent nucleoli and abundant eosinophilic cytoplasm, arranged in tubular, trabecular, and micropapillary patterns, infiltrating the dermis and resembling invasive ductal carcinoma of the breast (Fig. 3). IHC analysis showed diffuse expression of GATA3 and ER, with no reactivity for mammaglobin (Fig. 3). Based on these findings and in the absence of a prior history of breast cancer and negative breast-specific imaging, the tumor was favored as a primary from anogenital mammary-like glands. Tumor mass measured 4.7 cm in greatest dimension with 18 mm depth of invasion and lymphovascular invasion was present. The superior margin was focally positive for carcinoma and the tumor was 9 mm from the deep resection margin. Left sentinel node was negative for carcinoma. We established a diagnosis of invasive mammary-like adenocarcinoma of the vulva with breast TNM stage of T2N0M0 and vulvar FIGO stage IB grade 2. Somatic testing was significant for a BRCA1 mutation (variant p.S770, c.2309C>G). Germline testing revealed microsatellite stability and was negative for any BRCA or TP53 mutation.

Patient was referred to radiation given the positive margin but ultimately declined any adjuvant treatment. At 9 month follow up she remains without evidence of disease with confirmatory PET CT at 6 months showing no active disease. She has since developed a recurrence of her multiple myeloma which is being monitored by her primary hematologic oncologist.

## 3 Discussion

Mammary-like adenocarcinoma of the vulva typically presents as a fixed subcutaneous lesion with irregular borders, sometimes with ulceration, discoloration, or bleeding, with definitive diagnosis made based upon histopathology (Mansour et al., 2023, Morais et al., 2022, Irvin et al., 1999). The diagnostic criteria for mammary-like adenocarcinoma of the vulva include an invasive pattern resembling breast carcinoma, primarily localized in the subcutaneous tissue without direct involvement of the skin epithelium (Kazakov et al., 2011). Additional criteria include the presence of carcinoma in situ or non-neoplastic mammary tissue adjacent to the tumor, as well as presence of estrogen and/or progesterone receptors and immunohistochemical breast markers (Morais et al., 2022). Ductal carcinoma is the most common histological subtype, but others mirroring those in breast include mucinous, lobular, tubulo-lobular, mixed ductal-lobular, and secretory carcinoma (Mansour et al., 2023, Lopes et al., 2018, Morais et al., 2022, Buitrago-Flechas et al., 2021). Benign breast pathologies found in the vulva include fibrocystic changes, fibroadenomas, intraductal papillomas, or Phyllodes tumors (Irvin et al., 1999, Buitrago-Flechas et al., 2021).

Differential diagnosis for mammary-like gland carcinoma includes primary breast metastatic adenocarcinoma, extramammary Paget’s disease, sweat gland adenocarcinoma, and Bartholin gland adenocarcinoma (Morais et al., 2022). Immunohistochemical staining is crucial in establishing a diagnosis and differentiating mammary-like adenocarcinoma from other vulvar adenocarcinomas (Hu and Tiesinga, 2024). Extramammary Paget disease can be excluded using PAS-diastase, CEA, and GCDFP-15 staining, while metastases from gynecological and gastrointestinal origins are distinguished by PAX8 and CK20 expression, markers uncommon in mammary-like adenocarcinomas (Hu and Tiesinga, 2024). Other staining that is helpful in the diagnosis and management of mammary-like gland carcinoma of the vulva includes CK7, GATA3, ER, PR, and Her2/neu (Buitrago-Flechas et al., 2021). While estrogen and progesterone receptor positivity can be considered a diagnostic criterion, triple negative mammary-like gland carcinoma of the vulva has also been reported (Morais et al., 2022). Similar to breast carcinoma, mammary-like adenocarcinoma of the vulva can be classified into luminal A, luminal B, HER2-enriched, and basal-like subtypes (Tessier-Cloutier et al., 2017). In a prior case series, the majority of patients exhibited a luminal B pattern (3/7) with strong ER positivity, two patients exhibited HER-2 enriched, one exhibited luminal A pattern with low ER positivity, and one exhibited a basal-like pattern with triple-negative receptor staining (Tessier-Cloutier et al., 2017). Our patient exhibited a luminal A pattern and genomic testing confirmed presence of BRCA1 mutation not reproducible on germline testing. The presence of a BRCA1 tumoral mutation supports the idea that obtaining genomic testing may be useful in selecting potentially actionable therapeutic targets. It remains unclear if these patients can respond to a targeted therapeutic such as a Poly(ADP-ribose) polymerase (PARP) inhibitor, which has been widely associated with clinical responses in patients with BRCA mutations across solid tumors.

Existing literature has suggested managing primary mammary carcinoma of the vulva with the same approach as breast cancer given similar histology and behavior (Irvin et al., 1999, Buitrago-Flechas et al., 2021, Baykal et al., 2015). When confirmatory vulvar biopsy is obtained, a thorough metastatic workup should be performed, including mammography for evaluation for a breast primary cancer (Morais et al., 2022, Ananthula et al., 2020). Initial treatment typically involves surgical management with radical resection (Buitrago-Flechas et al., 2021, Ananthula et al., 2020). Lymph node evaluation by inguinal node dissection or sentinel node biopsy can be performed (Mansour et al., 2023, Ananthula et al., 2020). Our case shows sentinel lymph node detection using ICG dye is feasible in this disease.

Staging is based on the tumor, node, and metastasis system for breast or vulvar cancer, but optimal staging for treatment and prognosis has not been established (Mansour et al., 2023). Following surgery, observation, radiation, chemotherapy, and hormonal therapy have all been reported in the literature in varying combinations (Mansour et al., 2023, Irvin et al., 1999, Buitrago-Flechas et al., 2021, Ananthula et al., 2020, Wu et al., 2024).

A prior review that included 53 cases of vulvar mammary-like gland carcinoma explored a myriad of treatments, 30 % underwent lymphadenectomy, 33 % had radiation, 32 % received chemotherapy, and 35 % were given hormonal therapy (Buitrago-Flechas et al., 2021). Varying cytotoxic regimens have been utilized including combinations of taxanes, anthracyclines, and alkylating agents (Lopes et al., 2018, Niakan et al., 2021, Morais et al., 2022, Irvin et al., 1999). For patients with estrogen receptor positivity, hormonal therapy can be used for adjuvant treatment, utilizing selective estrogen receptor modulators such as tamoxifen or aromatase inhibitors (Lopes et al., 2018, Irvin et al., 1999, Wu et al., 2024). Patients with Her2 amplification have been treated with trastuzumab (Lopes et al., 2018, Niakan et al., 2021). CDK4/6 inhibitors have been used in metastatic disease (Ananthula et al., 2020). While surgery is usually the initial step, one report described use of neoadjuvant carboplatin, docetaxel, trastuzumab, and pertuzumab with no residual carcinoma identified at the time of vulvectomy (Niakan et al., 2021).

Though prognostic data is limited, reports suggest mammary-like gland adenocarcinoma of the vulva is prone to metastasis with spread pattern similar to breast cancer (Irvin et al., 1999). In addition to local recurrence, lung, bone, and brain metastases have been reported (Niakan et al., 2021, Baykal et al., 2015). Although our patient continues without evidence of disease, she has established surveillance with serial imaging given high risk of recurrence previously reported.

## 4 Conclusion

Mammary-like gland adenocarcinoma of the vulva is a rare but aggressive neoplasm that presents diagnostic challenges due to its resemblance to breast carcinoma. An accurate diagnosis relies on thorough histopathological evaluation and immunohistochemical profiling to differentiate it from other primary and metastatic tumors. Melanocytic skin types pose a unique set of differences regarding detection, diagnosis and potential genomic variations. Radical resection and sentinel lymph node assessment is feasible in selected cases. Ongoing histochemical and genomic assessments are warranted to further guide optimal therapeutic options.

## CRediT authorship contribution statement

**Lilla Markel:** Writing – original draft, Writing – review & editing. **Lidys Rivera:** Writing – original draft, Writing – review & editing. **A. Ordobazari:** Writing – original draft, Writing – review & editing. **Ardeshir Hakam:** Writing – review & editing. **Wilfredo Lorenzo:** Writing – review & editing. **Mitchel S. Hoffman:** Writing – review & editing. **Robert M. Wenham:** Writing – review & editing. **Monica Avila:** Conceptualization, Writing – original draft, Writing – review & editing.

## Declaration of competing interest

The authors declare that they have no known competing financial interests or personal relationships that could have appeared to influence the work reported in this paper.
